# Serum Erythropoietin level in anemia of elderly with unclear etiology

**DOI:** 10.1038/s41598-023-42806-7

**Published:** 2023-09-23

**Authors:** Ju Yong Seong, Dong-Yeop Shin, Ja Min Byun, Youngil Koh, Junshik Hong, Inho Kim, Sung-Soo Yoon

**Affiliations:** 1 Department of Internal Medicine, Seoul National University Hospital, Seoul National University College of Medicine, Seoul, Korea; 2 Center for Medical Innovation, Biomedical Research Institute, Seoul National University Hospital, Seoul, Korea; 3 Cancer Research Institute, Seoul National University College of Medicine, Seoul, Korea

**Keywords:** Diagnostic markers, Anaemia

## Abstract

Anemia is a common condition, but its causes are often unclear, especially in elderly adults. Erythropoietin (EPO) levels are known to be elevated in myelodysplastic syndrome and hematologic malignancies, but decreased in chronic benign anemia. This study aimed to investigate whether EPO levels could be used to identify underlying bone marrow diseases including malignancies, among elderly anemic patients with unclear etiology. This single centered retrospective study included patients presented with isolated anemia and had their EPO levels measured at their first visit. Patients were divided into two groups: bone marrow disease and benign etiologic anemia, based on observation and bone marrow test results. Out of 1180 patients reviewed, 81 patients with anemia of unclear etiology were identified, including 67 with benign anemia and 14 with bone marrow disease. Statistically significant difference in EPO levels between these two groups (*P* < 0.001) were observed. The receiver operating characteristic curve analysis showed that an EPO cut-off value of 36.4 mU/mL had a sensitivity and specificity of 92.8% and 94.0% for detecting underlying bone marrow disease, respectively. We suggest measuring serum EPO levels can aid in the early detection of benign anemia from bone marrow disease, including malignancies, with high sensitivity and specificity.

## Introduction

The prevalence of anemia is high in elderly adults, accounting nearly 12% in community setting, 47% in nursing home, 40% in hospitalized patients^1^. Anemia poses potential dangers in elderly patients such as frailty, decreased physical performance, cognitive decline, and increased hospitalization risks^2–4^. Elderly anemic patients can be broadly divided into three categories: nutritional deficiency (including iron, folate, vitamin B12 deficiency), anemia of inflammation (AI), and anemia of unknown etiology (AUE)^5^. AI is also known as anemia of chronic disease, which includes heterogenous conditions such as solid malignancies, chronic inflammation, infection and autoimmune diseases^6^. AI is diagnosed upon the presence of anemia with increased inflammatory biomarkers such as C-reactive protein, IL-6, or hepcidin^7^. AUE is a diagnosis of exclusion, used when no specific causes are found for anemia. Identifying the causes of anemia remains challenging, particularly in older anemic adults, where up to one-third of cases remain unknown^8–10^.

When patients present with anemia accompanied by cytopenia of granulocytic or megakaryocytic lineages, bone marrow tests are necessary to exclude hematologic malignancies such as myelodysplastic syndrome. In cases of isolated anemia without neutropenia or thrombocytopenia, initial approach starts with complete blood count tests, peripheral blood smear. Additional blood tests to measure iron profiles, vitamin B12/folate levels, serum creatinine, and monoclonal spike on serum protein electrophoresis are also evaluated. Obtaining clinical information from a patient's medical history is critical in diagnosing drug-induced anemia. If patients have chronic inflammatory conditions such as solid cancer, autoimmune disease, or chronic disease, AI can possibly be diagnosed. Still, AI is a diagnosis of exclusion^11^ and there is no specific diagnostic criterion for AI; the diagnosis can be arbitrary. Since AUE and AI are both diagnosis of exclusion and pathophysiology underlying the disease is multifactorial^10^, the diagnosis can be obscure and overlapping in some populations.

Anemia is more commonly observed in myelodysplastic syndrome (MDS) and other hematologic malignancies^12,13^ than thrombocytopenia or neutropenia^14^. Anemia is present even during the pre-diagnostic period of MDS^15^, making it possible that some individuals with AUE may have hidden MDS. Additionally, both MDS and anemia are prevalent in older adults, making detection of hidden MDS and AUE (or AI) challenging. Bone marrow tests can be used to differentiate MDS in these contexts, nevertheless, bone marrow tests are invasive and associated with complications, causing anxiety and reluctance in patients to undergo them^16,17^. Previous studies have shown that erythropoietin (EPO) levels are decreased in AUE^18–20^ and AI^19^ and elevated in MDS^19,21^ as well as in other hematologic malignancies^22^. We hypothesize that EPO levels could serve as a marker for detecting hidden bone marrow disease, including hematologic malignancies, in patients with AI or AUE.

## Results

### Baseline characteristics

**Figure 1:**
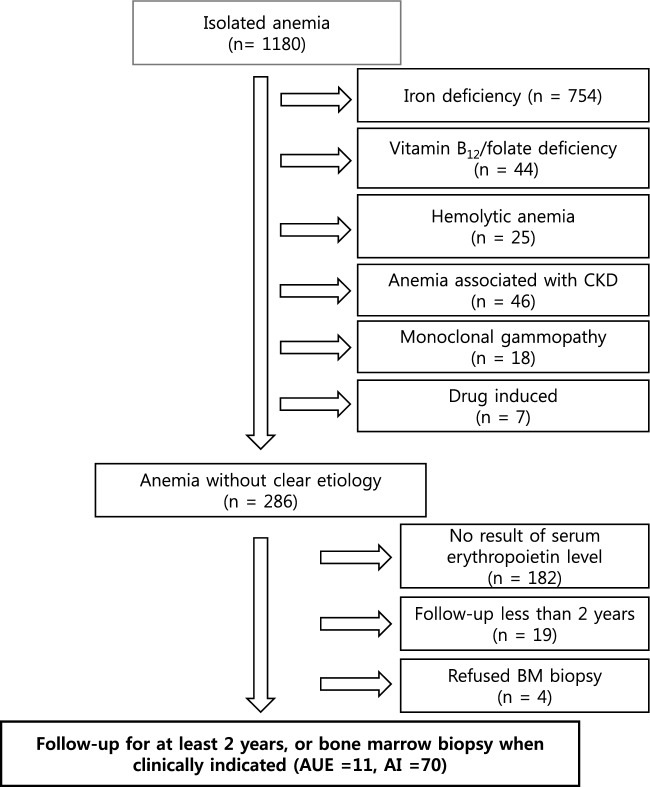
Study flowchart. Of the 1180 patients referred for isolated anemia, 286 were classified as having anemia of unclear etiology; data from 81 of these patients were analyzed. CKD, chronic kidney disease; BM, bone marrow; AI, anemia of inflammation; AUE, anemia of unknown etiology.

We retrospectively analyzed data from 1180 patients who presented at the hematology department with anemia without neutropenia or thrombocytopenia during the study period. Among them, 754, 44, 25, 46, 18, and 7 patients had IDA, megaloblastic anemia with vitamin B12 or folate deficiency, hemolytic anemia, anemia associated with chronic kidney disease, anemia associated with monoclonal gammopathy, and drug-induced anemia, respectively (Fig. 1). A total of 286 patients had anemia without a clear etiology, of which 182 were excluded due to a lack of EPO data, and an additional 19 were excluded due to loss to follow-up within 2 years. Four patients developed cytopenia during the follow-up period and were recommended for a bone marrow test, which they refused; consequently excluding them from this study.

Subsequently, data from a total of 81 patients (71 patients with AI and 10 patients with AUE) were included for analysis. Patients who did not experience anemia progression and were not diagnosed with either neutropenia or thrombocytopenia during the follow-up period were determined to have benign etiology anemia. Patients who showed newly developed bi-pancytopenia, progression of their condition, or evidence of malignancy were recommended to undergo bone marrow tests. Of the 25 patients who underwent bone marrow tests, two were diagnosed with acute myeloid leukemia, seven with myelodysplastic syndrome (MDS), three with pure red cell aplasia (PRCA), and eleven with anemia of benign etiology (no evidence of malignancy). Patients diagnosed with hematologic malignancy or PRCA via bone marrow biopsy were categorized into the bone marrow disease group, while the remaining patients were categorized into the anemia of benign etiology group (including both AI and AUE).

### Erythropoietin level difference in bone marrow disease associated anemia

**Figure 2:**
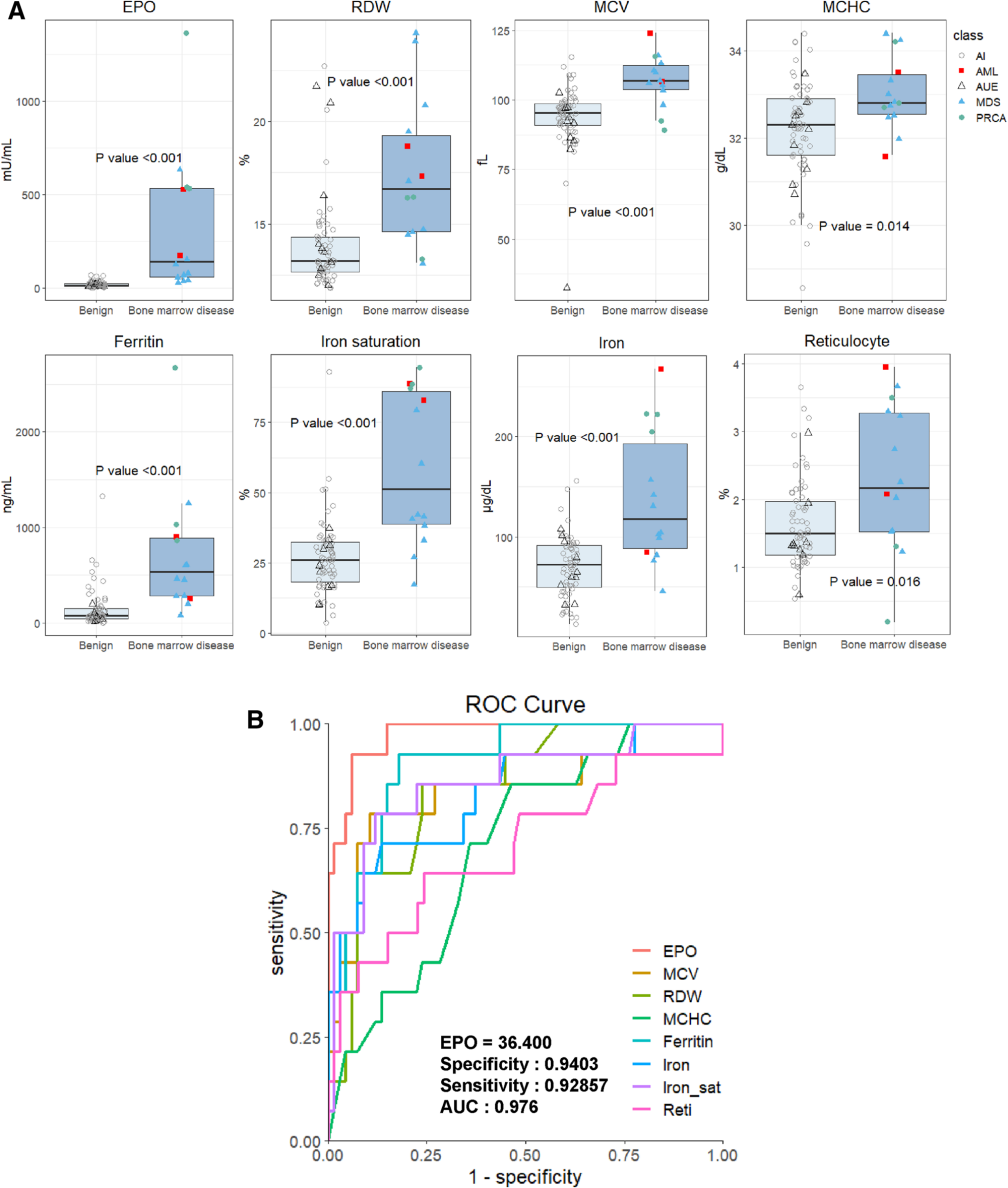
Erythropoietin is elevated in hidden bone marrow disease. (**A**) Box plot of Erythropoietin, red cell distribution width, mean corpuscular volume, ferritin, serum iron, and transferrin saturation, reticulocyte values in the bone marrow disease group and benign etiology anemia. (**B**) Receiver operating characteristic curve (ROC). The ROC curve was obtained with varying Erythropoietin (EPO) (Red), mean corpuscular volume (MCV) (yellow), red cell distribution width (RDW) (yellow green), mean corpuscular hemoglobin concentration MCHC (green), ferritin (cyan), iron (blue), transferrin saturation levels (iron_sat, purple), and reticulocyte (Reti, pink). The area under curve of EPO-curve is 0.976.

Statistically significant differences between the anemia of benign etiology group and bone marrow disease group were detected in EPO level (*P* < 0.001), red cell distribution width (RDW) (*P* < 0.001), mean corpuscular volume (MCV) (*P* < 0.001), Mean Corpuscular Hemoglobin Concentration (MCHC) (*P* = 0.014), ferritin (*P* < 0.001), transferrin saturation (*P* < 0.001), serum iron level (*P* < 0.001), and reticulocyte (*P* = 0.016) (Table 1). Box plots illustrating the differences are presented in Fig. 2A. The mean EPO level in the bone marrow disease group was approximately 20-fold higher than that in the benign etiology anemia group (16.7 vs. 310.2 mU/mL).

**Table 1:** Characteristics of patients with anemia of unknown etiology.

	Anemia of benign etiology (N = 67)	Anemia of bone marrow disease (N = 14)	*P*-value^a^
Mean (95% confidence interval)			
Age, years	73.1 (75.9–70.29)	75.5 (83.1–68.0)	0.5237
**Sex**	*N (%)*		
Male	19 (28)	6 (43)	0.3444^c^
Female	48 (72)	8 (57)	
Hemogram	*Mean (95% confidence interval)*		
WBC (10^3^/μL)	6.23 (6.63–5.83)	6.39 (7.35–5.44)	0.7312
Hemoglobin (g/dL)	9.52 (9.79–9.26)	8.6 (9.53–7.67)	0.5236
Platelet (10^3^/μL)	233.1 (254.7–211.6)	217.7 (268.8–166.5)	0.3329
MCV (fL)	94.0 (96.7–9.14)	106.9 (112.2–101.5)	< 0.001
MCHC (g/dL)	32.2 (32.5–31.9)	33.0 (33.5–32.5)	0.014
RDW (%)	13.9 (14.5–13.4)	17.4 (19.5–15.3)	< 0.001
Reticulocyte (%)	1.66 (1.82–1.50)	2.32 (2.96–1.68)	0.016
EPO (mU/mL)	16.7 (19.9–13.5)	310.2 (528.1–92.3)	< 0.001
Iron status			
Ferritin, (ng/mL)	145.2 (195.1–95.3)	708.7 (1090.3–327.0)	< 0.001
Iron (μg/dL)	70.2 (77.6–62.9)	138.7 (177.3–100.0)	< 0.001
Iron saturation (%)	26.8 (30.0–23.5)	58.6 (74.3–42.9)	< 0.001
TIBC (μg/dL)	282.1 (297.6–266.6)	263.0 (290.7–235.3)	0.142
CRP (mg/dL)	0.42 (0.62–0.22)	0.58 (1.30–0)	0.613
Creatinine (mg/dL)	0.98 (1.04–0.91)	0.83 (0.95–0.70)	0.060
BM biopsy^b^(%)	*N (%)*		
Yes	11 (16)	14 (100)	< 0.001^c^
No	56 (84)	0 (0)	

WBC: white blood cell, MCV: mean corpuscular volume, MCHC: mean corpuscular hemoglobin concentration, RDW: red cell distribution width, EPO: erythropoietin, TIBC: total iron binding capacity, LDH: lactate dehydrogenase, CRP: C-reactive protein, BM: bone marrow.

^a^*P*-value obtained using the Mann–Whitney U-test (except for sex and BM biopsy).

^b^Number of patients.

^c^*P*-value obtained using Fisher’s exact test.

^d^The lower 5% of 95% confidence interval showed − 0.58, where CRP level only exists in positive value; hence, we substituted it with zero.

The ROC analysis demonstrated that an EPO level of 36.4 mU/mL had the highest sensitivity (92.8%) and specificity (94.0%) for detecting bone marrow disease (Fig. 2B). At the EPO threshold of 25.0 mU/mL, all patients with bone marrow disease were correctly identified. The area under the ROC curve for EPO was 0.976.

**Figure 3:**
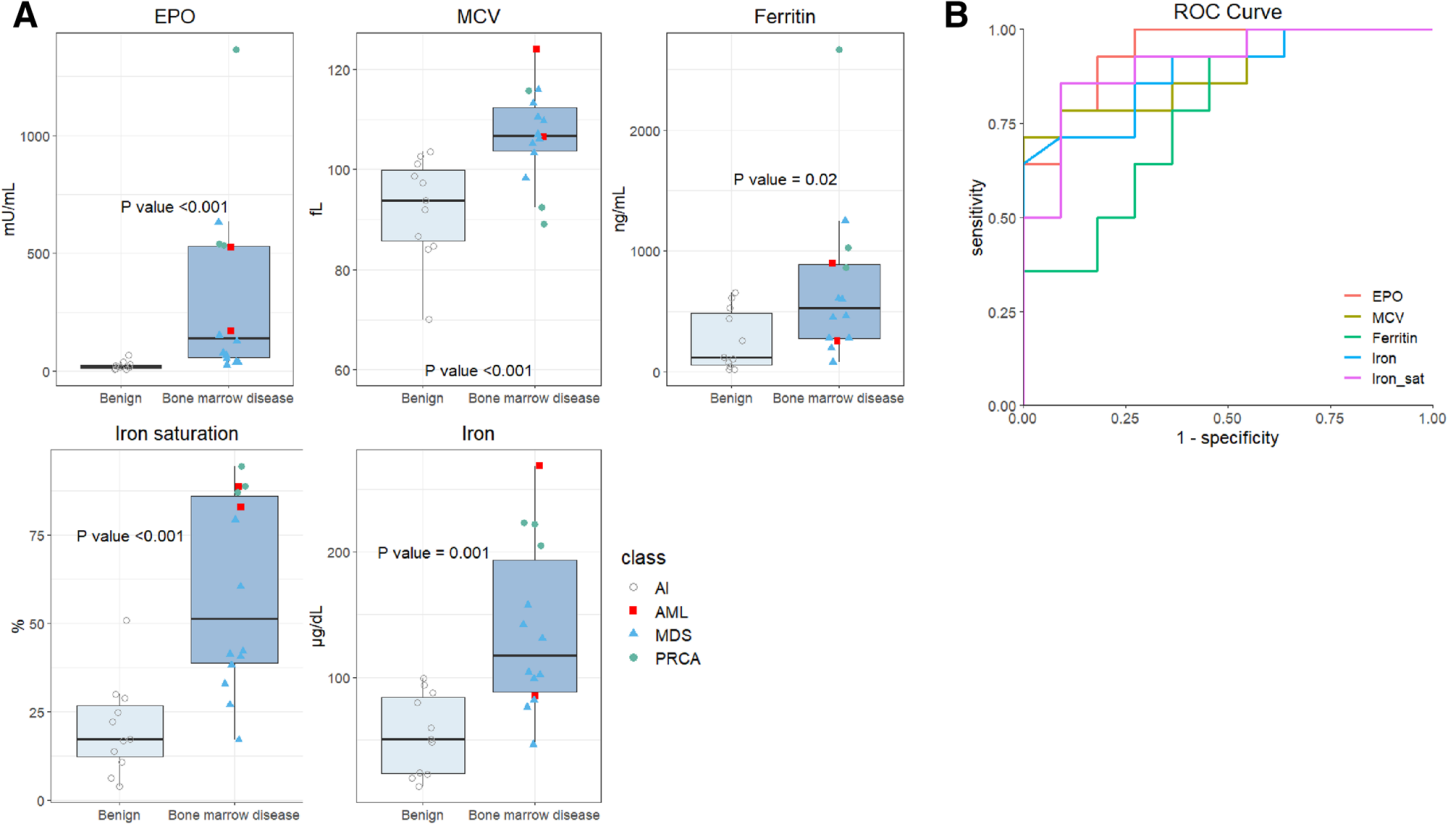
(**A**) Box plot of Erythropoietin, mean corpuscular volume, ferritin, serum iron, and transferrin saturation in the bone marrow disease and benign etiology anemia, with bone marrow test confirmed. (**B**) ROC curve, Erythropoietin (EPO) (Red), mean corpuscular volume (MCV) (yellow), ferritin (cyan), iron (blue), transferrin saturation (iron_sat, purple). Area under curve of EPO curve is 0.941.

A subgroup analysis was conducted on patients who underwent a bone marrow biopsy, including 14 patients with bone marrow disease and 11 patients with benign etiology anemia. Statistically significant differences were found between the groups in EPO level (*P* < 0.001), MCV (*P* < 0.001), ferritin (*P* = 0.021), serum iron level (*P* = 0.001), and transferrin saturation (*P* < 0.001), as shown in Table 2 and Fig. 3A. The ROC curve for EPO demonstrated a sensitivity of 92.8% and specificity of 81.8% at an EPO level of 36.7 mU/mL, with an area under the curve of 0.941 (Fig. 3B).

**Table 2:** Characteristics of patients with confirmed BM biopsy.

	Anemia of benign etiology (N = 11)	Anemia of bone marrow disease (N = 14)	*P*-value^a^
Mean (95% confidence interval)			
Age, years	73.1 (78.7–67.5)	75.5 (83.1–68.0)	0.5279
Sex	*N (%)*		
Male	4 (36)	6 (43)	1
Female	7 (64)	8 (57)	
Hemogram	*Mean (95% confidence interval)*		
WBC (10^3^/μL)	5.69 (7.01–4.83)	6.39 (7.35–5.44)	0.3809
Hemoglobin (g/dL)	8.73 (9.58–7.89)	8.6 (9.53–7.67)	0.8692
Platelet (10^3^/μL)	197.9 (244.4–151.5)	217.7 (268.8–166.5)	0.8508
MCV (fL)	92.2 (99.07–85.4)	106.9 (112.2–101.5)	< 0.001
MCHC (g/dL)	32.1 (33.1–31.2)	33.0 (33.5–32.5)	0.1247
RDW (%)	15.2 (16.4–13.9)	17.4 (19.5–15.3)	0.1061
Reticulocyte (%)	1.92 (2.54–1.30)	2.32 (2.96–1.68)	0.3242
EPO (mU/mL)	23.1 (35.2–10.9)	310.2 (528.1–92.3)	< 0.001
Iron status			
Ferritin, (ng/mL)	260.7 (429.2–92.1)	708.7 (1090.3–327.0)	0.0210
Iron (μg/dL)	54.5 (76.0–33.1)	138.7 (177.3–100.0)	0.001
Iron saturation (%)	20.5 (29.4–11.6)	58.6 (74.3–42.9)	< 0.001
TIBC (μg/dL)	243.0 (291.2–194.7)	263.0 (290.7–235.3)	0.3172
CRP (mg/dL)	1.11 (1.84–0.374)	0.58 (1.30–0)	0.1319
Creatinine (mg/dL)	0.93 (1.12–0.74)	0.83 (0.95–0.70)	0.3959

WBC: white blood cell, MCV: mean corpuscular volume, MCHC: mean corpuscular hemoglobin concentration, RDW: red cell distribution width, EPO: erythropoietin, TIBC: total iron binding capacity, LDH: lactate dehydrogenase, CRP: C-reactive protein, BM: bone marrow.

^a^*P*-value obtained using the Mann–Whitney U-test (except for sex).

^b^Number of patients.

^c^*P*-value obtained using Fisher’s exact test.

^d^The lower 5% of 95% confidence interval showed − 0.58, where CRP level only exists in positive value; hence, we substituted it with zero.

## Discussion

In this study, we found that EPO was notably decreased in the benign etiology anemia group (including AI and AUE), reflecting relative erythropoietin production deficiency underlying its pathophysiology^23^. Anemia of bone marrow diseases showed elevated EPO, probably due to an impaired response to EPO in the bone marrow, which in turn may leads to the production of more EPO. This phenomenon is similarly seen in MDS, where elevated serum EPO predicts an impaired response to erythropoiesis-stimulating agents^24,25^. Among 81 patients initially sorted as anemia with unclear etiology, 14 patients were found to have bone marrow disease, which accounts for 17% of the initial anemia with unclear etiology. This prevalence is similar to previous studies reporting that 17.2% of patients with unexplained anemia were highly possibly MDS^8^ and 17.9% of hematologic diseases were found among AUE^26^. Our findings show that EPO levels are decreased in AI or AUE while increased in bone marrow disease-associated anemia, suggesting its role in differentiating bone marrow disease in elderly anemic patients with unclear etiology.

Besides a difference in EPO, RDW and MCV were higher in bone marrow disease group, which was consistent with previous reports^27,28^, though RDW was not significantly different in the subgroup analysis, indicating that it may not be a reliable marker for detecting bone marrow disease in some cases. We suggest macrocytic anemia without clear etiology with elevated EPO level should be suspected of bone marrow disease in elderly patients. Iron panel -ferritin, iron, transferrin saturation- showed significant difference between the groups. Previous reports have shown that serum ferritin levels are elevated in AML and MDS at diagnosis, and that a higher ferritin level is associated with a poor prognosis^29,30^. Additionally, dysregulated iron metabolism is a known feature of certain bone marrow disorders, including MDS and AML^31^, with potential role of leukemia progression^32,33^. The differences in iron panels between the groups in our study might reflect altered iron metabolism in bone marrow diseases; however, further research would be needed to fully understand the underlying mechanisms behind these differences.

Previous studies have reported the presence of anemia years before overt MDS^15^. One patient in our study showed elevated EPO level with idiopathic cytopenia of undetermined significance (ICUS) confirmed by bone marrow biopsy, who later developed MDS within 8 months (Supplementary table 1). ICUS has been proposed as a potential precursor to MDS, with somatic mutations potentially driving its evolution^34,35^. Currently, there is no clinically useful biomarker to predict ICUS progression to MDS. Though the evidence is weak, our study suggests that EPO levels may serve as an early marker of erythroid lineage failure and could potentially predict progression from ICUS to MDS, although further research is needed to confirm these findings.

Our study has some limitations that should be taken into account when interpreting the results. The relatively small sample size may limit the power and precision of our findings, and being a single-center study may limit generalizability. Second, EPO is not a specific marker for bone marrow disease and can be influenced by other factors such as renal function, hypoxia, recent bleeding, or hemolysis. Therefore, the interpretation of elevated EPO levels should be made in the context of the patient's clinical presentation, excluding other factors for EPO elevation. Third, due to the retrospective nature of the study, we excluded 182 out of the total 286 patients with anemia without a clear etiology due to a lack of EPO data. The decision to test EPO levels in anemia patients was solely based on the treating physician's choice, as there were no specific predefined criteria for such testing in our center. Physicians may have been influenced by the severity of anemia or suspicion of MDS, which could have affected their decision to test EPO levels in the patients. This should be taken into account when interpreting the results.

Recently, next-generation sequencing (NGS) technology has emerged as a powerful tool for detecting clonal hematopoiesis, potentially offering valuable diagnostic insights for anemia with unclear etiology^36^. Thus, the future impact of using serum EPO may be limited in the era of NGS. However, the current cost of NGS remains high, making widespread application to every patient with anemia impractical. Testing EPO for screening bone marrow disease can still be a financially reasonable choice for many patients with anemia of unclear etiology. In conclusion, the present study provides preliminary evidence for the potential diagnostic utility of EPO levels in identifying underlying bone marrow disease among elderly anemic patients with unclear etiology.

## Methods

### Study design and data collection

We conducted a retrospective cohort study of patients referred to the Department of Hematology at Seoul National University Hospital for isolated anemia (anemia without other cytopenia) from January 1, 2017 to January 31, 2021. The study was approved by the Institutional Review Board of Seoul National University Hospital (IRB No. H-2105–043-1229). Waiver of informed consent was granted by the Institutional Review Board of Seoul National University Hospital. All methods were performed in accordance with the relevant guidelines and regulations. The study was performed in accordance with the Declaration of Helsinki. Data were extracted from the electronic medical records. The demographic and clinical characteristics of patients, including hemogram, iron status, vitamin B12/folate status, hemolysis, EPO level, creatinine level, serum protein electrophoresis, and C-reactive protein values, and bone marrow test findings, were reviewed.

### Definition

Anemia was defined when hemoglobin levels below 12.0 g/dL and 13.0 g/dL in women and men, respectively. Thrombocytopenia was defined as platelet counts below 150,000/μL and neutropenia was defined as an absolute neutrophil count of less than 1500/μL. Patients were excluded if they had concurrent neutropenia or thrombocytopenia. The diagnostic groups included iron deficiency anemia (IDA), nutrition deficiency anemia (vitamin B12 or folate deficiency), hemolysis, chronic kidney disease, monoclonal gammopathy, drug-induced anemia, and AUE. IDA was diagnosed when ferritin levels were below 15 ng/mL and transferrin saturation values were below 10%. Anemia with nutritional deficiency was defined as serum vitamin B12 levels below 200 pg/mL or folate levels below 3 ng/mL. Chronic kidney disease was diagnosed when estimated glomerular filtration rate was below 45 mL/min/1.73 m2 (calculated with the Chronic Kidney Disease Epidemiology Collaboration Eq. ^37^). Hemolysis was diagnosed when elevation of lactate dehydrogenase (> 280 U/L) or plasma Hb (> 5 mg/dL) were noted or haptoglobin was decreased (< 41 mg/dL) with relevant clinical evidence of hemolysis (e.g., prosthetic valve or schistocytes observed in peripheral blood smear). Monoclonal gammopathy was diagnosed with serum or urine electrophoresis. Drug-induced anemia was classified as anemia occurring within 2 months of recent use of cytotoxic drugs with clinical risks. AI was diagnosed when patients had a chronic inflammatory disease, including solid cancer, autoimmune disease, chronic infection, chronic obstructive pulmonary disease, and heart failure. AUE was diagnosed in patients that did not meet any of these diagnostic criteria. Patients with AI and AUE who had data of EPO levels at their first visit to the department of hematology and clinical follow-up data obtained over a period of more than 2 years were included in this study.

### Statistical analysis

Baseline hemogram, iron status, creatinine level, and EPO levels were compared between the anemia of benign etiology and bone marrow disease groups using the Mann–Whitney U test. Fischer’s exact test was used to compare binary data. *P*-values less than 0.05 were considered statistically significant. Statistical analysis and ROC (Receiver Operating Characteristic) curve was performed using R software (version 4.2.1) with ggplot2^38^, pROC^39^ packages and Microsoft Excel® (Microsoft Office 2013, Microsoft Corporation, Redmond, WA, USA).

### Supplementary Information

Supplementary Information.

## Data Availability

The data that support the findings of this study are available from the corresponding author upon reasonable request.
