# Supplementary material for: Serum Erythropoietin level in anemia of elderly with unclear etiology
Source: Sci Rep. 2023 Sep 23;13:15902. doi: 10.1038/s41598-023-42806-7 (PMC10517950; doi:10.1038/s41598-023-42806-7)
Supplement: Supplementary file 1 — Supplementary Information. [file 41598_2023_42806_MOESM1_ESM.pdf]

Title: Serum Erythropoietin level in anemia of elderly with unclear etiology.

Authors:

Juyong Seong[1], Dong-Yeop Shin[1,2,3*], Ja Min Byun[1,3], Youngil Koh[1,2,3], Junshik Hong[1,2,3], Inho Kim[1,3], Sung-Soo Yoon[1,3]

Affiliations

[1]Department of Internal Medicine, Seoul National University Hospital, Seoul National University College of Medicine, Seoul, Korea

[2]Center for Medical Innovation, Biomedical Research Institute, Seoul National University Hospital, Seoul, Korea

[3]Cancer Research Institute, Seoul National University College of Medicine, Seoul, Korea

* shindongyeop@snu.ac.kr

Supplementary table 1. ICUS patients who developed MDS

	Patient 1
**Age, years**	79
**Sex**	Female
**Hemogram**	
WBC ($10^3/\mu L$)	9.22
Hemoglobin (g/dL)	6.7
Platelet ($10^3/\mu L$)	185
MCV (fL)	106
MCHC (g/dL)	32.8
RDW (%)	14.6
**EPO** (mU/mL)	632
**Iron status**	
Ferritin, (ng/mL)	279.7
Iron (μg/dL)	99
Iron saturation (%)	40.6
TIBC (μg/dL)	269
**LDH** (IU/L)	-
**CRP** (mg/dL)	0.48
**Creatinine** (mg/dL)	1.01
**Initial diagnosis**	Anemia of unknown Etiology
**Initial BM**	Normal BM, ICUS
**Follow up BM**	MDS

WBC: white blood cell, MCV: mean corpuscular volume, MCHC: mean corpuscular hemoglobin concentration, RDW: red cell distribution width, EPO: erythropoietin, TIBC: total iron binding capacity, LDH: lactate dehydrogenase, CRP: C-reactive protein, BM: bone marrow, CKD: chronic kidney disease, ICUS: idiopathic cytopenia of undetermined significance, MDS: myelodysplastic syndrome

Initial BM biopsy was performed 1 month after initial laboratory evaluation, and follow-up BM biopsy was performed 8 months after initial [illegible]
